# Efficacy of different routes of vitamin B12 supplementation for the treatment of patients with vitamin B12 deficiency: A systematic review and network meta-analysis

**DOI:** 10.1007/s11845-023-03602-4

**Published:** 2024-01-17

**Authors:** Omar Ahmed Abdelwahab, Ahmed Abdelaziz, Sherein Diab, Ali khazragy, Toka Elboraay, Taha Fayad, Rehab Adel Diab, Ahmed Negida

**Affiliations:** 1 Medical Research Group of Egypt, Cairo, Egypt; 2 Faculty of Medicine, Al-Azhar University, Cairo, Egypt; 3 Faculty of Medicine, Ain Shams University, Cairo, Egypt; 4 Faculty of Medicine, Alexandria University, Alexandria, Egypt; 5 Faculty of Medicine, Zagazig University, Zagazig, Egypt; 6 Faculty of Oral and Dental Medicine, Sinai University, Sinai, Egypt; 7 Faculty of Medicine, Al-Azhar University for Girls, Cairo, Egypt; 8 School of Pharmacy and Biomedical Sciences, University of Portsmouth, Portsmount, UK; 9 Department of Global Health and Social Medicine, Harvard Medical School, Boston, MA USA

**Keywords:** Administration, Intramuscular, Oral, Sublingual, Vitamin B12

## Abstract

**Background:**

This systematic review and network meta-analysis aimed to evaluate the three different administration routes of vitamin B12: oral, intramuscular (IM), and sublingual (SL) routes.

**Methods:**

We searched four electronic databases (PubMed, Scopus, Web of Science, and Cochrane CENTRAL Register of Controlled Trials). We included only comparative studies. We performed a frequentist network meta-analysis to measure network estimates for the relative outcomes. Moreover, we conducted a pairwise meta-analysis using a random effect model to obtain direct estimates for outcomes. All outcomes were continuous, and the relative treatment effects were pooled as mean difference (MD) with 95% confidence intervals.

**Results:**

Thirteen studies were included in the meta-analysis, with a total of 4275 patients. Regarding increasing vitamin B12 levels, the IM route ranked first, followed by the SL route (MD = 94.09 and 43.31 pg/mL, respectively) compared to the oral route. However, these differences did not reach statistical significance owing to the limited number of studies. Regarding the hemoglobin level, the pooled effect sizes showed no difference between all routes of administration that could reach statistical significance. However, the top two ranked administration routes were the oral route (78.3) and the IM route (49.6).

**Conclusion:**

All IM, oral, and SL routes of administration of vitamin B12 can effectively increase the level of vitamin B12 without significant differences between them, as thought previously. However, the IM route was the top-ranked statistically but without clinical significance. We found no significant difference among studied administrated routes in all other CBC parameters and homocysteine levels.

**Graphical Abstract:**

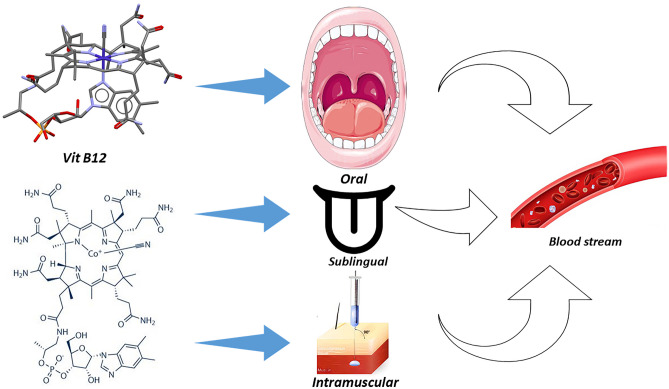

**Supplementary Information:**

The online version contains supplementary material available at 10.1007/s11845-023-03602-4.

## Introduction 

**Fig. 1:**
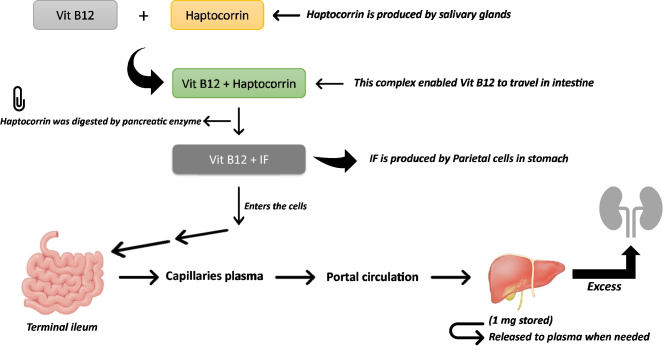
Vitamin B12 absorption, transport and metabolism

Vitamin B12 is a water-soluble vitamin that may be derived from foods, including fish, meat, dairy products, and cereals that have been fortified. It can also be taken in supplement form. After being extracted by gastric acid, it travels to the terminal ileum, where it is co-absorbed along with the intrinsic factor, an enzyme from the stomach’s parietal cells (Fig. 1) [1, 2]. Vitamin B12 is essential for neurologic function, red blood cell creation, and DNA synthesis and is a coenzyme for three primary biochemical conversions: homocysteine to methionine, 5-methyltetrahydrofolate to tetrahydrofolate, and methylmalonic acid to succinyl coenzyme A [1, 2].

Reduced serum vitamin B12 levels (< 200 ng per mL [148 pmol per L]) are considered vitamin B12 deficiency [1, 2] and are associated with reduced hematopoietic and epithelial cell proliferation, elevated levels of methylmalonic acids, and propionic that impact the myelin sheaths of nerve fibers, and elevated serum homocysteine, a contributing factor for cardiovascular disorders [3]. Blood manifestations related to the deficiency in vitamin B12 are anemia (megaloblastic macrocytic), leukopenia, and thrombocytopenia; moreover, a paradoxical thrombocytosis may occur [4, 5]. In neuropsychiatry, it may cause areflexia, peripheral neuropathy, olfactory impairment, gait irregularities, proprioception, and vibratory sensation loss, cognitive problems (including dementia-like manifestations and psychosis), and irritability. In the gastrointestinal, it may cause glossitis [5–8]. So, the treatment of vitamin B12 deficiency is devastating.

There are different routes for the administration of any vitamin. Oral and intramuscular (IM) vitamin B12 are the most common routes for treating vitamin B12 deficiency, and several studies have evaluated their efficacy [9, 10]. However, in the case of vitamin B12 malabsorption, irrespective of the cause of malabsorption, the intramuscular route is preferred [11, 12]. According to the British Society for Hematology recommendations, for individuals with severe insufficiency and malabsorption syndromes, IM vitamin B12 delivery is preferable; however, oral replacement therapy may be recommended for patients with asymptomatic, moderate illness who have no concerns about absorption or compliance [13].

The sublingual (SL) route of vitamin B12 administration has been evaluated as an alternative route to oral and IM routes, especially in the case of vitamin B12 malabsorption [14–16], but there is no sufficient data to consider this route in the guidelines. This systematic review and network meta-analysis aimed to assess the efficacy of those three different routes of administration of vitamin B12.

## Methods

For this research, we used the same format that the PRISMA statement recommends for systematic reviews and meta-analyses [17]. Every stage was carried out in a manner entirely consistent with the Cochrane Handbook of Systematic Reviews and Meta-analyses of Interventions [18].

### Eligibility criteria

Studies were included in our review if they satisfied the following criteria:

- Population: studies on patients with vitamin B12 deficiency
- Intervention and comparator: studies where the experimental and control groups received vitamin B12 through any of these routes of administration: SL, IM, and oral
- Outcome: studies reporting at least any of the following serum levels: vitamin B12, folate, hemoglobin, hematocrit, mean corpuscular volume, plasma homocysteine, urine methylmalonic acid, leukocyte, and platelets
- Study design: comparative studies whose design was controlled trials with patients allocated to receive SL, IM, or oral vitamin B12 in a random or non-random allocation manner. We considered both blinded and open-label studies. We also included controlled prospective and retrospective observational studies with extreme caution; these studies were separated from randomized controlled trials in subgroups and were only considered in calculating the pooled effect size if their results were consistent with randomized controlled trials. In case of discrepancy between randomized controlled trials and observational studies, the results highlighted this, and the outcomes of randomized controlled trials were prioritized

Studies for which complete full texts were not accessible, studies not published in English, studies whose data were unsuitable for extraction and analysis, and studies that were described as abstracts only or thesis were all excluded.

### Information sources and search strategy

We performed a comprehensive search of four electronic databases (PubMed, Scopus, Web of Science, and Cochrane Central Register of Controlled Trials) from inception until May 7, 2022, using the following query: (B 12, Vitamin OR Vitamin B12 OR B12, Vitamin OR Cyanocobalamin OR Cobalamins OR Cobalamin OR Eritron OR Vitamin-B12 OR Hydroxocobalamin OR Deltavit B12) AND (Sublingual OR tongue OR Oral OR Intravenous OR IV OR Inhalation OR Buccal OR Cutaneous OR Mucosal OR Parenteral OR Subcutaneous OR SC OR Intramuscular OR IM OR Intranasal). The search was carried out by O.A.A.

Furthermore, the references of the included studies were manually searched for any potentially eligible studies. The detailed search strategy and results for each database are presented in Supplementary 1.

### Selection process

Five authors (S.D., A.K., R.A.D., T.E., and T.F.) separately screened the titles and abstracts of all identified articles to determine their relevance to this meta-analysis. The second step involved screening the full-text articles of the included abstracts to determine the final eligibility for meta-analysis. Duplicates were excluded using Endnote (Clarivate Analytics, PA, USA). The discussion was used to settle any disagreements.

### Data collection process and data items

Five reviewers (S.D., A.K., R.A.D., T.E., and T.F.) collected data onto a standardized data extraction sheet. Extracted information focused on four primary areas: (1) summary of the included studies (including study ID, title, study design, country, inclusion criteria, exclusion criteria, interventions, dose, sample size, treatment duration, and the main findings); (2) study population characteristics (including age, sex body mass index, serum level of vitamin B12, hematocrit, hemoglobin, and mean corpuscular volume (MCV) levels); (3) risk of bias domains according to the study design; and (4) outcome measures (the primary outcomes were the serum levels of vitamin B12 and hemoglobin and the secondary outcomes includes the levels of MCV, homocysteine, platelets count, and white blood cells count). The discussion was used to settle any disagreements.

### Assessing the risk of bias in the individual studies

We independently evaluated the quality of each included study by two authors (S.D. and T.E.). The Cochrane assessment tool was used for randomized clinical trials (ROB2) [19]. Newcastle Ottawa scale (NOS) was used to assess the risk of bias for observational studies [20]. For non-randomized controlled trials, we used the Cochrane ROBINS-I tool [21]. A third author (O.A.A.) solved any disagreements.

### Statistical analysis

We measured network estimates for the related outcomes using a frequentist network meta-analysis of aggregate data. The evident heterogeneity in the intervention comparison effects across studies was accommodated using the random effect model as a framework. Moreover, we conducted a pairwise meta-analysis using a random effect model to obtain direct estimates for outcomes. The transitivity assumption was examined to determine if patient and research characteristics were sufficiently comparable across comparisons. Additionally, using a loop-specific methodology, we assessed the consistency assumption locally in a closed loop [22]. The surface under the cumulative ranking (SUCRA) was used to rank the intervention’s hierarchy in the network model; then, we estimated the mean ranks [23]. A comparison-adjusted funnel plot was used to explore the potential publication bias [23].

We used a three-level hierarchical network meta-analysis to incorporate the exchangeability between different study designs to predict an effect estimate for each study design individually [24]. Thus, this model allows strength to be borrowed within the different classes of study designs, strengthening interference and potentially reducing the uncertainty around each study design and consequently increasing the ability to inform decision-making frameworks. All data of this approach were pooled as Cohen’s *d* with 95% confidence intervals.

All outcomes of interest were continuous, and the relative treatment effects were pooled as mean difference (MD) with 95% confidence intervals. All analyses were done in STATA version 17 using the network command.

## Results

### Literature search results

**Fig. 2:**
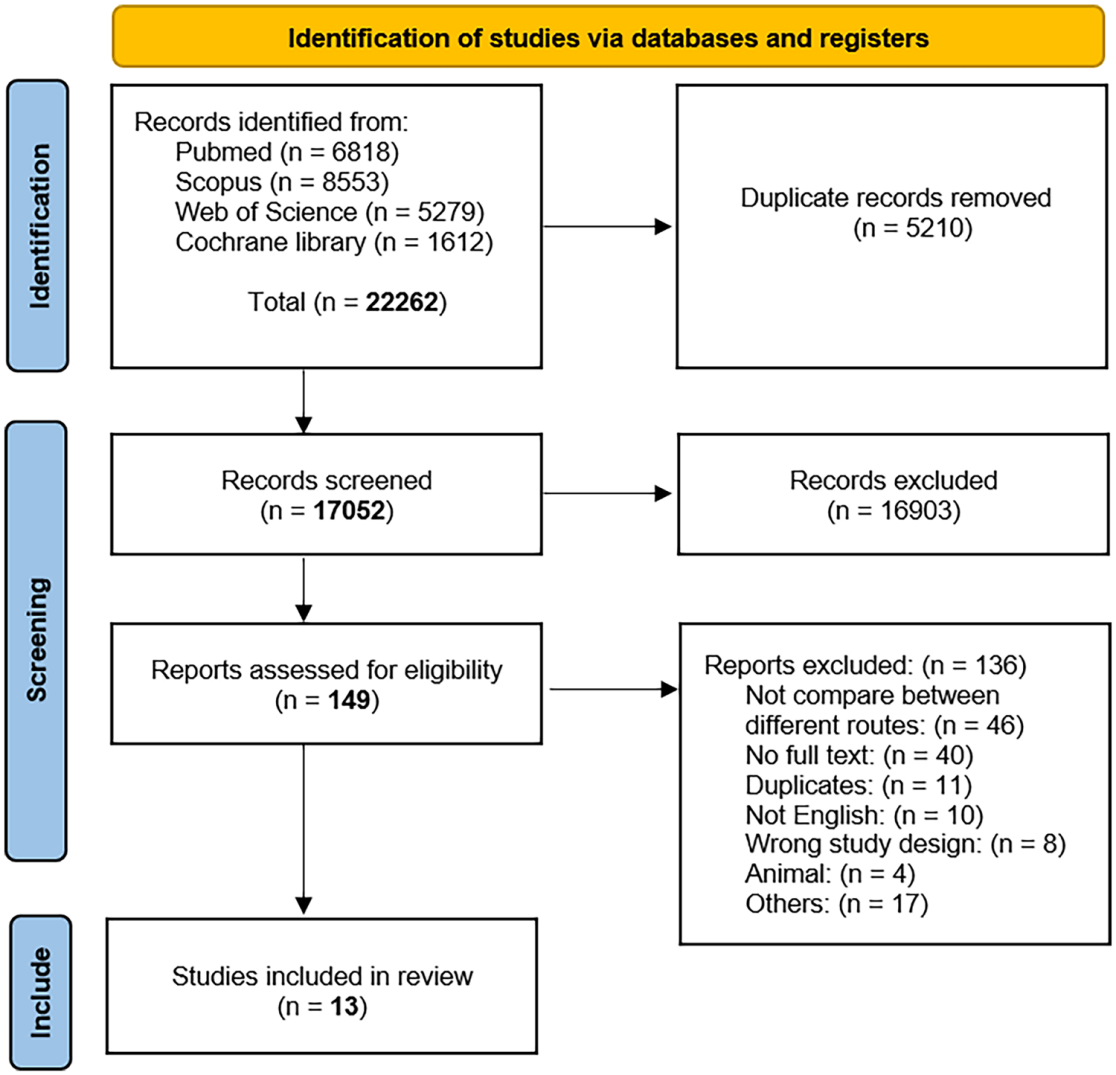
PRISMA flow diagram of studies’ screening and selection

Our literature search process retrieved 22,262 records. Five thousand two hundred ten duplicates were removed using Endnote, and 17,052 were screened for title and abstract. One hundred forty-nine articles were qualified for full-text screening after being subjected to title and abstract screening. The meta-analysis comprised 13 of these investigations. No further papers were included after manually searching the references of the listed studies. Figure 2 illustrates the PRISMA flow diagram of the study recruitment process.

### Characteristics of the included studies

Thirteen studies were included in the meta-analysis, with a total of 4275 patients. In all studies, patients were assigned to receive either oral, SL, or IM vitamin B12. A summary of the included studies and the characteristics of the population in each study are provided in Tables 1 and 2, respectively. According to the Cochrane ROB 2 tool, four studies had some concerns, two had high, and one had a low risk of bias. According to ROBINS-I checklists, one study had a high risk of bias, and one had a moderate risk of bias. Two studies had good quality, and one had fair quality, according to the NOS tool (Supplementary 2).

**Table 1:** Summary of included studies

**Study ID**	**Title**	**Design**	**Country**	**Inclusion criteria**	**Exclusion criteria**	**Interventions**	**Dose**	**No. of participants**	**Treatment duration**	**Main findings**
Adachi et al. [25]	Enteral vitamin B12 Supplements Reverse postgastrectomy B12 Deficiency	Non-randomized controlled clinical trial	Japan	Patients with vitamin B12 deficiency who underwent gastrectomy	NR	Oral	500 µg/day	3	3 months	Both oral and IM vitamin B12 significantly improve serum vitamin B12 levels and correct the deficiency
							750 µg/day	10		
							1500 µg/day	5		
						IM	500 µg/2 months	4	6 months	
							500 µg/month	6		
Bensky et al. [26]	Comparison of sublingual vs. intramuscular administration of vitamin B12 for the treatment of patients with vitamin B12 deficiency	Retrospective analysis of data	Israel	Adults who were prescribed vitamin B12, irrespective of their vitamin B12 levels	Folate-deficient (3 ng/mL) patients. phenobarbital, methotrexate, carbamazepine, mesalazine, valproic acid, sulfamethoxazole, and phenytoin induce megaloblastic anemia. Non-vitamin B12d anemic patients. Patients with vitamin B12 or excipient hypersensitivity	SL	1 mg	3451	6 months	SL vitamin B12 significantly improves serum vitamin B12 levels more than IM vitamin B12
						IM		830		
Bolaman et al. [27]	Oral versus intramuscular cobalamin treatment in megaloblastic anemia: a single-center, prospective, randomized, open-label study	RCT	Turkey	Patients with a serum vitamin B12 of less than 160 pg/mL, megaloblastic anemia, and an MCV of more than 94 fL	Patients who have had vomiting and/or diarrhea, drink more than 40 g of alcohol per day, cannot give informed consent, have a history of cancer, folate deficiency, cannot swallow pills, or are taking medicine that might interrupt the folate metabolism as methotrexate. Women who are pregnant, might be pregnant, or are breastfeeding	Oral	1000 μg /day for 10 days, once a week for 4 weeks, and once a month forever	26	90 days	Oral was as effective as IM vitamin B12 treatment. When compared to IM therapy, oral was more tolerated and less costly
						IM		34		
Castelli et al. [28]	Comparing the efficacy and tolerability of a new daily oral vitamin B12 formulation and intermittent intramuscular vitamin B12 in normalizing low cobalamin levels: a randomized, open-label, parallel-group study	RCT	USA	Patients with overall good health and appropriate renal function whose clinical laboratory testing revealed vitamin B12 deficiency, with serum vitamin B12 < 350 pg/mL	Patients on the treatment of vitamin B12 deficiency, on antacids, cannot tolerate the oral medication, had hypersensitivity to vitamin B12, or have folate deficiency	oral	1000 μg/day	24	90 days	Both the oral and IM routes were effective in achieving normal levels of vitamin B12 in all patients investigated (100%)
						IM	1000 μg/day	26		
Kuzminski et al. [29]	Effective treatment of cobalamin deficiency with oral cobalamin	RCT	USA	Patients with low vitamin B12 and high serum methylmalonic acid and total homocysteine	Patients who live outside of the local geographic region of Bassett Hospital, are unable to provide informed consent, refuse to participate, or have a life-threatening disease	Oral	2000 μg/day	18	4 months	Oral was more effective than IM in raising vitamin B12 level at 2 months
						IM	1000 μg/day on days 1, 3, 7, 10, 14, 21, 30, 60, and 90	15		
Metaxas et al. [30]	Early biomarker response and patient preferences to oral and intramuscular vitamin B12 substitution in primary care: a randomized parallel-group trial	RCT	Switzerland	Adult patients with vitamin B12 concentration < 200 pmol/l and were able to give written informed consent	Patients on the treatment of vitamin B12 deficiency, dementia, with known genetic transcobalamin transport abnormalities, or a lack of written and/or spoken comprehension of German, French, Italian, or English	Oral	1000 μg/day	19	28 days	IM vitamin B12 was highly statistically significant more than oral vitamin B12 in raising vitamin B12 and holotranscobalamin levels and decreasing homocysteine levels
						IM	1000 μg/week	18		
Orhan Kiliç et al. [31]	Sublingual methylcobalamin treatment is as effective as intramuscular and per oral cyanocobalamin in children aged 0–3 years	Retrospective Cohort	Turkey	Patients between 0 and 3 years of age and with vitamin B12 values are < 300 ng/L were included in the study between January 2017 and August 2020	NR	Oral cyanocobalamin group	1000 μg ampules of oral cyanocobalamin	89	Every day for the first week, every other day for 2 weeks, 2 days/week for 2 weeks, followed by once a week for three months	Oral, SL, and IM vitamin B12 administration all significantly improve the vitamin B12 level
						SL Methylcobalamin group	1 puff of sublingual methylcobalamin 1000 μg	46	Every day for the first week, every other day for 2 weeks, 2 days/week for 2 weeks, followed by once a week for three months	
						IM cyanocobalamin group	100 μg (1/10) of 1000 μg ampule intramuscular cyanocobalamin	23	Every day for the first week, every other day for 2 weeks, 2 days/week for 2 weeks, followed by once a week for three months	
Sanz-cuesta et al. [10]	Oral versus intramuscular administration of vitamin B12 for vitamin B12 deficiency in primary care: a pragmatic, randomized, non-inferiority clinical trial (OB12)	RCT	Spain	Patients were 65 years of age or older, with a serum concentration of vitamin B12 of < 211 pg/mL	NR	Orally	1 mg/day in weeks 1–8 and 1 mg/week in weeks 9–52	140	52 weeks	Oral administration was no less effective than IM administration at 8 weeks
						Intramuscular	1 mg of vitamin B12 on alternate days in weeks 1–2, 1 mg/week in weeks 3–8, and 1 mg/month in weeks 9–52	143	52 weeks	
Schijing et al. [32]	Efficacy of oral compared with intramuscular vitamin B-12 supplementation after Roux-en-Y gastric bypass: a randomized controlled trial	RCT	Netherlands	Patients diagnosed with a low value of vitamin B-12 (< 200 pmol/L)	Patients were excluded if there was an increase in their creatinine concentration (> 150 µmol/L) or liver enzymes (> 2 times the upper limit), if they had undergone previous gastrointestinal surgery other than RYGB, had a gastrointestinal disease or psychiatric issues, had used medication that influenced their bone metabolism, had already used a vitamin B-12 supplement other than the advised multivitamin supplements, or were pregnant	Orally	1000 μg of methylcobalamin orally, once daily, for 6 months	24	6 months	The efficacy of oral vitamin B12 supplementation was similar to that of injections
						Intramuscular	Loading dose injection of 2000 μg of hydroxocobalamin at baseline followed by bimonthly injections of 1000 μg of hydroxocobalamin up until 6 months	26	6 months	
Sezer et al. [33]	Comparison of the efficacy of parenteral and oral treatment for nutritional vitamin B12 deficiency in children	Non-randomized controlled clinical trial	Turkey	Children aged between 1 month and 18 years old with serum vitamin B12 levels under 300 pg/mL	Newborns, patients with chronic diseases, patients with a history of allergic reactions to vitamin B12, patients who were receiving micronutrient supplementation, and patients who failed to give consent	Orally	1000 mcg	82	1 month	Both oral and parenteral formulations were shown to be effective in normalizing vitamin B12 levels
						Intramuscular	1 ml; 1000 mcg	60	1 month	
Sharabi et al. [34]	Replacement therapy for vitamin B12 deficiency: comparison between the sublingual and oral route	RCT	Israel	Adult subjects with a serum cobalamin concentration < 138 pmol/L	NR	SL vitamin B12 group	500 μg	10	8 weeks	SL vitamin B12 and oral vitamin B12 both are effective in correcting serum vitamin B12 in patients with vitamin B12 deficiency
						Oral vitamin B12 group	500 μg	10	8 weeks	
						Oral vitamin B complex group	Two tablets, each containing 250 μg cobalamin, 100 mg thiamine, and 250 mg pyridoxine	10	8 weeks	
Strong et al. [35]	Sublingual vitamin B12 compared to intramuscular injection in patients with type 2 diabetes treated with metformin: a randomized trial	RCT	New Zeeland	Patients with type 2 DM who were being treated with metformin for 12 months or longer, and a screening serum vitamin B12 of < 220 pmol/L	Patients already on treatment for vitamin B12 deficiency (including over-the-counter vitamin supplementation containing vitamin B12), were anemic for another reason, had prior gastric surgery (e.g., gastric bypass), were pregnant or breastfeeding, reported past cobalamin allergy, or other reason in the judgment of the investigators as to why vitamin B12 could not be administered	SL vitamin B12 group	1 mg/day	19	3 months	Decreased serum vitamin B12 levels in patients with type 2 diabetes on long-term metformin treatment can be corrected through treatment with either hydroxocobalamin injections or methylcobalamin sublingual supplements
						IM vitamin B12 group	A single dose of 1 mg	15	3 months	
Tuğba-Kartal and Cağla-Mutlu [36]	Comparison of sublingual and intramuscular administration of vitamin B12 for the treatment of vitamin B12 deficiency in children	Retrospective study	Turkey	Patients with vitamin B12 deficiency (serum vitamin B12 level ≤ 200 pg/mL), aged 5–18 years, and between January 2017 and December 2019	Patients with chronic diseases that may affect hematologic parameters (sideroblastic anemia, thalassemia, aplastic anemia, etc.), folate deficiency, iron deficiency, renal disease, using drugs that may affect the absorption of vitamin B12 (metformin, proton pump inhibitors, phenobarbital, etc.), and having missing data	IM cyanocobalamin	1000 mcg	47	Every other day for 1 week, then weekly for 3 weeks	SL cyanocobalamin and methylcobalamin are as effective as IM cyanocobalamin in correcting serum vitamin B12 levels and hematologic abnormalities in children with vitamin B12 deficiency
						SL cyanocobalamin	1000 mcg SL tablet	43	Once daily for 7 days, then every other day for 3 weeks	
						SL methylcobalamin	1000 mcg 10 mL spray	39	Once daily for 7 days, then every other day for 3 weeks	

*SL* sublingual, *IM* intramuscular, *RCT* randomized controlled trial

**Table 2:** Baseline characters of included studies

**Study ID**	**Interventions**	**No. of participants**	**Age**		**Sex (males)**		**BMI**		**Hematocrit (%)**		**Hemoglobin levels (g-dl)**		**MCV levels (fL)**		**Serum vitamin B12 levels (pg/mL) **	
			**Mean**	**SD**	**Number**	**percent**	**Mean**	**SD**	**Mean**	**SD**	**Mean**	**SD**	**Mean**	**SD**	**Mean**	**SD**
Adachi et al. [25]	Oral 500 µg/day	3	NR		23	74.1	NR		NR		NR		NR		132	26
	Oral 750 µg/day	10													151	25
	Oral 1500 µg/day	5													112	28
	IM 500 μg/2 months	4													102	51
	IM 500 μg/month	6													127	51
Bensky et al. [26]	SL	3451	NR		NR		NR		NR		13.2	1.45	88.73	5.37	298	155
	IM	830									13.01	1.62	88.77	6.14	234	102
Bolaman et al. [27]	Oral	26	60	15	16	61.5	NR		NR		8.4	2.1	112.3	11.4	72.9	54.8
	IM	34	64	10	17	50					8.3	2.3	114.8	10.9	70.2	59.1
Castelli et al. [28]	Oral	24	52.6	15.27	5	20.8	30.9	7.09	NR		NR		NR		285.5	54.27
	IM	26	53.8	15.68	6	23.1	31.8	7.67							262	54.61
Kuzminski et al. [29]	Oral	18	72	11	6	33.3	NR		37.6	6.2	NR		100	12	93	42
	IM	15	71	15	2	13.3			39.5	2.9			102	11	95	92
Metaxas et al. [30]	Oral	19	47.3	17.8	6	31.6	27.3	7	NR		13.8	1.31	NR		214.1	37.1
	IM	18	51.5	19.6	8	44.4	25.6	4.2			13.6	1.8			222.2	27.2
Orhan Kiliç et al. [31]	Oral	89	1.08	0.58	45	50.5	NR		NR		11.6	1.2	NR		201.1	63.2
	SL	46	1.08	0.58	21	45.6					11.3	2.1			187	49.2
	IM	23	1	0.66	10	43.5					12.1	1.2			176.1	64.2
Sanz-cuesta et al. [10]	Oral	140	74.2	5.8	53	37.9	NR		42.4	4	NR		92.1	6.7	173.1	27.3
	IM	143	76.2	6.7	65	45.5			41.9	4.2			94.3	7.4	166.4	32.6
Schijing et al. [32]	Oral	24	46	11.9	5	19	32.9	4.5	NR		NR		NR		223.58	75
	IM	26	41.6	9.8	8	33	29.2	4.4							206.9	58.4
Sezer et al. [33]	Oral	82	NR		NR		NR		35.3	3.3	11.7	1.2	78.8	6.5	183.5	47
	IM	60							34.6	4.2	11.6	1.5	79.7	7.9	175.5	42.5
Sharabi et al. [34]	SL	10	44.5	14.7	8	80	NR		NR		NR		NR		127.4	40.7
	Oral	10	50.2	15.1	7	70									146.38	23.04
	Oral vitamin B complex	10	49.7	11.6	7	70									132.8	18.97
Strong et al. [35]	SL	19	63.7	7.8	11	57.9	NR		NR		NR		NR		230.7	52.9
	IM	15	64.9	6.8	9	60									225.9	49.47
Tuğba-Kartal and Cağla-Mutlu [36]	IM	47	12.7	5.1	26	55.3	NR		NR		10.1	1.3	82.6	10.8	147.5	37.7
	SL	82	12.1	4.1	40	48.8					10.3	1.5	81.7	6.9	141.7	38.5

*SL* sublingual, *IM* intramuscular

### Primary outcomes

#### Vitamin B12

**Fig. 3:**
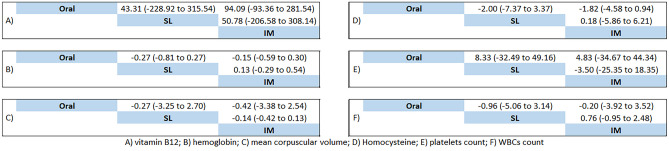
Network estimates of treatment effect on each outcome

The network of treatment comparisons for vitamin B12 included three active individual nodes (Fig. S1; top panel)***.*** Each node represents a different administration route; the oral administration route was the most well-connected intervention with all other interventions directly linked to it; therefore, it has been used as the reference for comparison. Figure 3A shows network estimates of treatment effect on vitamin B12 levels for different route administrations compared with the oral route. Network meta-analysis showed that either the IM route (MD 94.09 pg/mL, 95% CI [− 93.36 to 281.54]) or the SL route (MD 43.31 pg/mL, 95% CI [− 228.92 to 315.54]) compared to the oral route did not reach a significant difference to increase vitamin B12 levels. According to SUCRA values, the top-ranked intervention for increasing levels of vitamin B12 was the IM route (74.2), followed by the SL route (48.4) (Table 3(A)).

**Table 3:** SUCRA of vitamin B12 and hemoglobin

A				B			
**SUCRA of vitamin B12**				**SUCRA of Hb**			
**Treatment**	**SUCRA**	**PrBest**	**Mean Rank**	**Treatment**	**SUCRA**	**PrBest**	**Mean Rank**
IM	74.2	57.5	1.5	oral	78.3	68.2	1.4
SL	48.4	32.6	2	IM	49.6	22.3	2
Oral	27.4	9.9	2.5	SL	22.1	9.5	2.6

We performed a three-level hierarchical model to investigate the pooled effect sizes according to the study design in which there was no significant difference between the IM and the oral routes compared to the SL route (*P* = 0.31, 0.16, respectively). There was a significant difference between the oral and the IM routes (Cohen’s d − 0.74, 95% CI [− 1.06 to − 0.43]; *P* < 0.001). The pooled effect sizes were not homogenous (*I*^2^ > 50%); Fig. S2.

#### Hb

Five studies comprising 3730 patients reported Hb. The network diagram included three individual nodes, (Fig. S1; bottom panel). Each node represents a different drug intervention, in which the oral route was the well-connected route of administration with all other routes directly linked to it.

Figure 3B shows network estimates of treatment effect on Hb levels for different administration routes compared to the oral route. Pooled effect sizes showed no difference between all routes of administration to increase Hb without substantial statistical evidence (Fig. 3B). According to SUCRA values, the top two ranked administration routes were the oral route (78.3) followed by the IM route (49.6) (Table 3(B)).

The three-level hierarchical model showed no significant difference among all comparisons of administration routes; Cohen’s *d* was 0.07 for IM vs SL routes, 0.06 for oral vs IM routes, and 0.22 for oral vs SL routes. The pooled effect size was homogenous (*I*^2^ = 0%) (Fig. S3).

#### Secondary outcomes

Five included studies comprising 3605 patients reported change in MCV, four studies comprising 140 patients reported change in homocysteine levels, four studies comprising 3588 patients reported change in platelets count, and only three studies with 3430 patients reported change in WBC count.

Figures S4 and S5 show networks of routes of administration for secondary outcomes. Figure 3C–F summarize the results for secondary outcomes. Network meta-analysis showed no evidence of differences among all possible comparisons for secondary outcomes (MCV, homocysteine levels, platelet counts, and WBC counts). Supplementary Table 5 presents SUCRA values. However, none of the three-level hierarchical models showed any significance among all possible comparisons of administration routes (Figs. S6-S9).

## Discussion

### Significance of the study

To our knowledge, this is the most comprehensive network meta-analysis comparing the efficacy of SL, IM, and oral routes of administration of vitamin B12 in patients with vitamin B12 deficiency. The significance of this paper is not only to compare the three routes of administration but also to evaluate them and determine which route is the best to administrate the drug. The study also opens the door for more future research about the administration routes of vitamin B12 and other vitamins.

### Summary of the findings

The current article evaluated 13 studies, of which eight were randomized clinical trials comparing different administration routes of vitamin B12 in patients with vitamin B12 deficiency. A total of 4275 patients with vitamin B12 deficiency were included in the final analysis. We found that irrespective of the route of vitamin B12 administration, serum vitamin B12 levels were increased. When comparing the different routes, the top-ranked route for increasing levels of vitamin B12 was the IM route, followed by the SL route. However, this difference has no clinical significance.

Interestingly, we found no significant difference among studied administrated routes in all other CBC parameters such as Hb, MCV, platelets count, WBC count, and homocysteine level. Given the fact that vitamin B12 levels were increased insignificantly among all routes, the preference of the administrated route should be referenced to the advantages and disadvantages of each route as well as the patient situation, which will be decided according to the physician’s opinion.

**Fig. 4:**
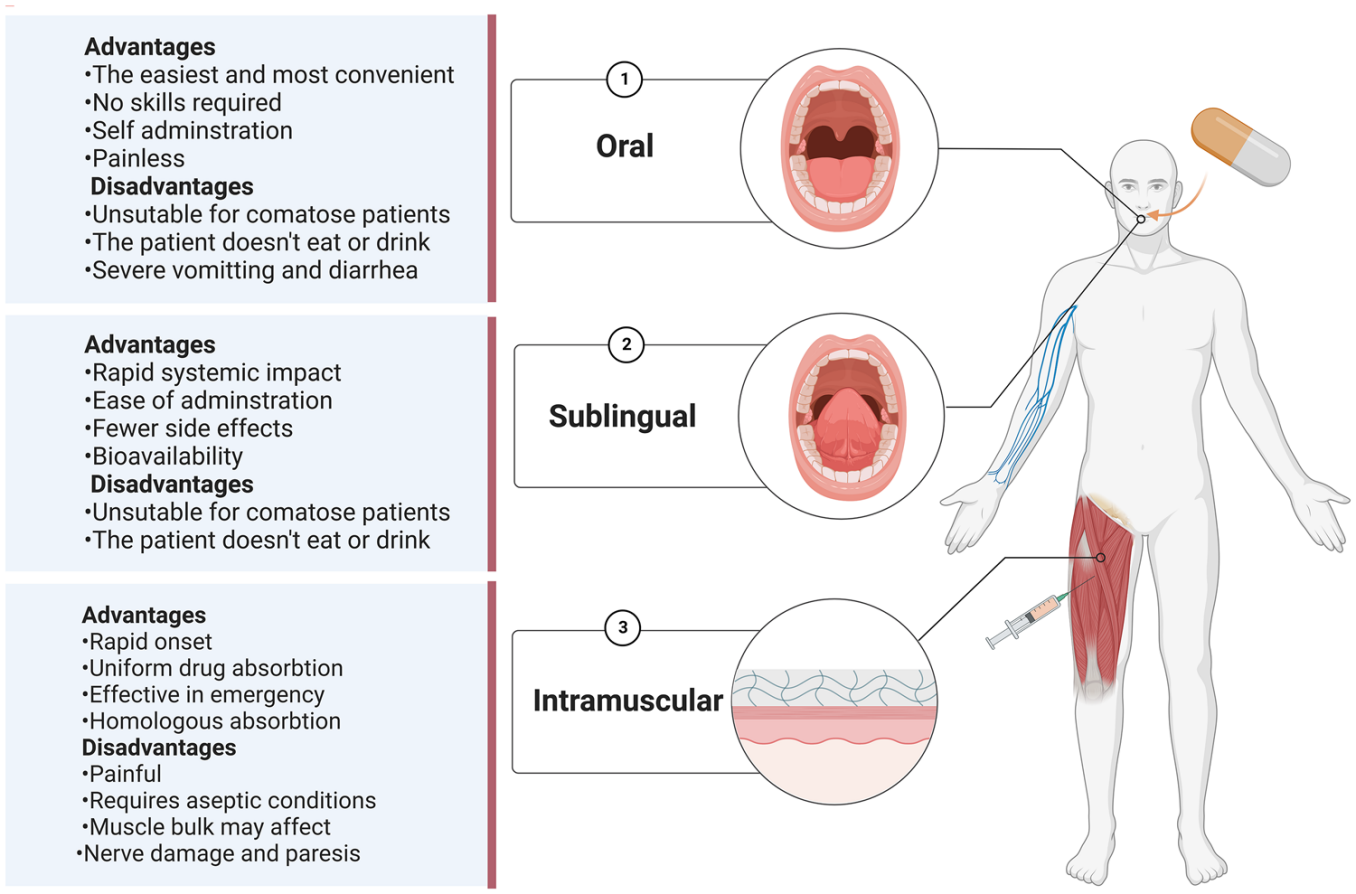
Advantages and disadvantages of each route of administration

We highlighted the summary of the advantages and disadvantages of each route in (Fig. 4) [37–48], as treatment decision depends on the patient’s condition and the physician’s opinion. The details about the advantages and disadvantages of each route are present in Supplementary 4.

### Sublingual vs intramuscular and oral routes of administration

A previous systematic review by Cochrane compared the effectiveness of oral versus IM routes of vitamin B12 administration. It showed that both effectively improve serum vitamin B12 levels for people suffering from vitamin B12 deficiency. However, the dosage of oral vitamin B12 might be a clinical factor that affects this outcome [9]. This contributes to the absorption mechanism of oral vitamin B12, which does not depend only on intrinsic factors, but the absorption can be done through passive diffusion. Passive diffusion accounts for 1.2% of overall absorption, with little effect on bioavailability in patients with pernicious anemia or gastro-duodenal resection [49, 50].

As our results showed, there is no difference between them in terms of efficacy, but in terms of patients’ tolerance, advantages, and disadvantages of each route, the sublingual route is preferred over IM and oral routes. SL vitamin B12 is as adequate as IM and oral vitamin B12. However, the SL route of administration has more advantages and fewer disadvantages than IM and oral routes. So, we recommend using SL vitamin B12 rather than oral and IM, especially in patients who do not tolerate IM injection and patients who need prolonged vitamin B12 supplementation, such as patients with long-term metformin use and patients with pernicious anemia or with gastro-duodenal resection [51–54]. In terms of cost, the IM route is higher in cost than the SL and oral routes [2, 55].

We recommend future research directions to determine when to start with SL, IM, or oral vitamin B12 in different situations.

### Implications in practice

As there is no apparent clinical significance, the treatment should depend on the patient’s condition. We advise physicians to examine every patient carefully to exclude every cause that may lead to malabsorption and then choose the most appropriate route. SL route could be used in patients with pernicious anemia or malabsorption causes. However, the oral route cannot be excluded as it did not depend on intrinsic factors only, as the simple diffusion (without the need for intrinsic factors) is now considered another mechanism for the absorption of vitamin B12 [49, 50].

Before this meta-analysis, the IM route was generally the preferred route, but our results showed that no route is usually preferred, and SL and oral routes should be considered comparable alternative routes.

### Strength points and limitations

Based on our knowledge, this is the most updated meta-analysis on this topic, including all available evidence based on our inclusion criteria of including only controlled studies, either RCTs or controlled observational studies, to reach the highest accessible quality of evidence from the available evidence found in the literature. We are the first meta-analysis comparing the different administration routes of vitamin B12, and the first meta-analysis includes the SL route of administration in the analysis.

The limitations of this work are that we included RCTs, non-RCTs, and observational studies, which may lower the overall quality of evidence of the included studies. We cannot find the full text of one study which seems to be included. The head-by-head comparison between the three interventions was made only in one paper of the included studies. Additionally, there was variability between the included studies in the follow-up duration, which may cause heterogeneity in the results obtained.

## Conclusion

All IM, oral, and SL routes of administration of vitamin B12 can effectively increase the level of vitamin B12 without significant differences between them, as thought previously. However, the IM route was the top-ranked statistically, followed by the SL and then the oral routes, but without clinical significance. We found no significant difference among studied administrated routes in all other CBC parameters such as Hb, MCV, platelets count, WBC count, and homocysteine level. 

### Supplementary Information

Below is the link to the electronic supplementary material.

Supplementary Material 1 (DOCX 899 KB)

## Data Availability

The datasets used and/or analyzed during the current study are available as MS Excel files (.xlsx) and RevMan file (.rm5) from the corresponding author upon reasonable request.

## Declarations

### Protocol registration

The protocol of this study was not registered.
