# Supplementary material for: Efficacy of different routes of vitamin B12 supplementation for the treatment of patients with vitamin B12 deficiency: A systematic review and network meta-analysis
Source: Ir J Med Sci. 2024 Jan 17;193(3):1621–39. doi: 10.1007/s11845-023-03602-4 (PMC11128391; doi:10.1007/s11845-023-03602-4)
Supplement: Supplementary file 1 — Supplementary Material 1 (DOCX 899 KB) [file 11845_2023_3602_MOESM1_ESM.docx]

## Search strategy for each database and its results:

**Pubmed:**

Strategy: (B 12, Vitamin OR Vitamin B12 OR B12, Vitamin OR Cyanocobalamin OR Cobalamins OR Cobalamin OR Eritron OR Vitamin-B12 OR Hydroxocobalamin OR Deltavit B12) AND (Sublingual OR tongue OR Oral OR Intravenous OR IV OR Inhalation OR Buccal OR Cutaneous OR Mucosal OR Parenteral OR Subcutaneous OR SC OR Intramuscular OR IM OR Intranasal)

Results: **6818**

**Web of Science:**

Strategy: (B 12, Vitamin OR Vitamin B12 OR B12, Vitamin OR Cyanocobalamin OR Cobalamins OR Cobalamin OR exitron OR Vitamin-B12 OR Hydroxocobalamin OR deltanil B12) (All Fields) and (Sublingual OR tongue OR Oral OR Intravenous OR IV OR Inhalation OR Buccal OR Cutaneous OR Mucosal OR Parenteral OR Subcutaneous OR SC OR Intramuscular OR IM OR Intranasal) (All Fields)

Results: **5279**

**Scopus:**

Strategy: ( TITLE-ABS-KEY ( ( "B 12, Vitamin" OR "Vitamin B12" OR "B12, Vitamin" OR "Cyanocobalamin" OR "Cobalamins" OR "Cobalamin" OR "Eritron" OR "Vitamin-B12" OR "Hydroxocobalamin" OR "Deltavit" ) ) AND TITLE-ABS-KEY ( ( "Sublingual" OR "tongue" OR "Oral" OR "Intravenous" OR "IV" OR "Inhalation" OR "Buccal" OR "Cutaneous" OR "Mucosal" OR "Parenteral" OR "Subcutaneous" OR "SC" OR "Intramuscular" OR "IM" OR "Intranasal" ) ) )

Results: **8553**

**Cochrane Library:**

Strategy: (B 12, Vitamin OR Vitamin B12 OR B12, Vitamin OR Cyanocobalamin OR Cobalamins OR Cobalamin OR Eritron OR Vitamin-B12 OR Hydroxocobalamin OR Deltavit B12) AND (Sublingual OR tongue OR Oral OR Intravenous OR IV OR Inhalation OR Buccal OR Cutaneous OR Mucosal OR Parenteral OR Subcutaneous OR SC OR Intramuscular OR IM OR Intranasal)

Results: **1612**

## Risk of bias assessment of the included studies:

Table 1 Risk of bias according to the ROBINS-I tool for the included studies

|  | Bias due to/in | | | | | | |  |
| --- | --- | --- | --- | --- | --- | --- | --- | --- |
|  | Confounding | Selection of participants into the study | Classification of interventions | Deviations from intended interventions | Missing data | Measurement of outcomes | Selection of the reported result | Overall bias |
| **Adachi 2000** | Low | Low | Low | No information | Low | Low | Moderate | Moderate |
| **Sezer 2018** | Serious | Serious | Moderate | Moderate | Low | Low | Low | Serious |

Table 2 Risk of bias according to the ROB 2 tool for the included studies

|  |  |  | Bias due to/in |  |  |  |
| --- | --- | --- | --- | --- | --- | --- |
|  | Randomization process | Deviations from intended interventions | Measurement of the outcome | Missing outcome data | Selection of the reported result | Overall bias |
| **Bolaman 2003** | Some concerns | Low | Low | Low | Low | Some concerns |
| **Castelli 2011** | Some concerns | Low | Low | Low | Low | Some concerns |
| **Kuzminski 1998** | Some concerns | Low | Low | Some concerns | Low | Some concerns |
| **Metaxas 2017** | Low | Low | Low | Low | Low | Low |
| **Sanz-cuesta 2019** | Low | Low | Low | Low | Low | Low |
| **Schijing 2018** | Some concerns | High | Low | Low | Low | High |
| **Sharabi, 2003** | High | Low | Low | Low | Low | High |
| **Strong, 2016** | Some concerns | Low | Low | Low | Low | Some concerns |

Table 3 Risk of bias according to the NOS tool for the included cohort studies

|  | Selection |  |  |  | Comparability | Exposure |  |  | Overall score |
| --- | --- | --- | --- | --- | --- | --- | --- | --- | --- |
|  | Representativeness of the exposed cohort | Selection of the non-exposed cohort | Ascertainment of exposure | Demonstration that the outcome of interest was not present at the start of the study | Comparability of cohorts on the basis of the design or analysis | Assessment of outcome | Was follow-up long enough for outcomes to occur | Adequacy of follow-up of cohorts |  |
| **Bensky, 2019** |  | * | * | * | ** | * | * | * | 8 (Good) |
| **Orhan Kiliç, 2021** |  |  | * | * | * | * |  |  | 4 (Fair) |
| **Tuğba-Kartal, 2020** |  | * | * | * | ** | * | * | * | 8 (Good) |

## Figures:

**Figure S1:**


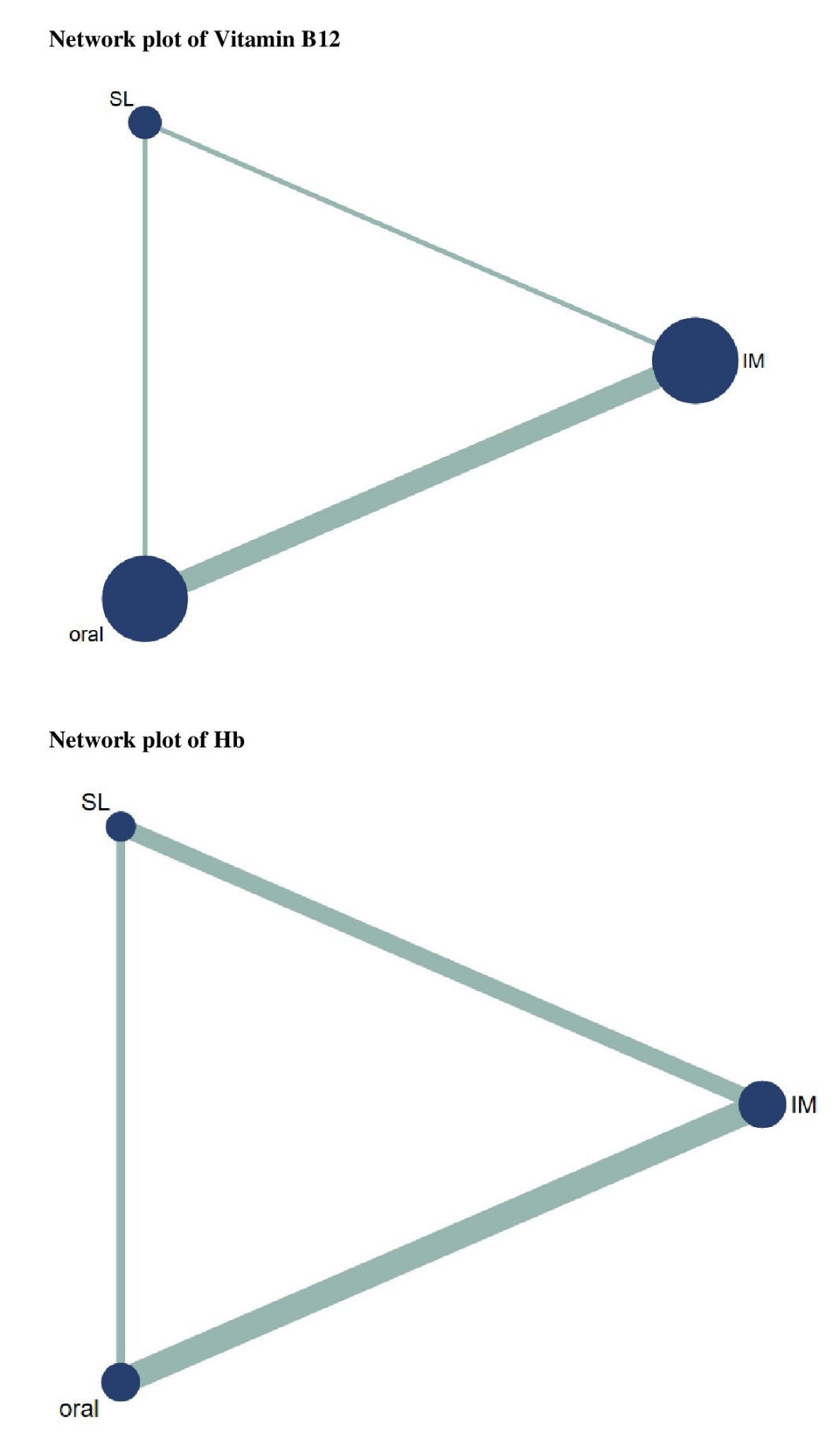


**Figure S2:**


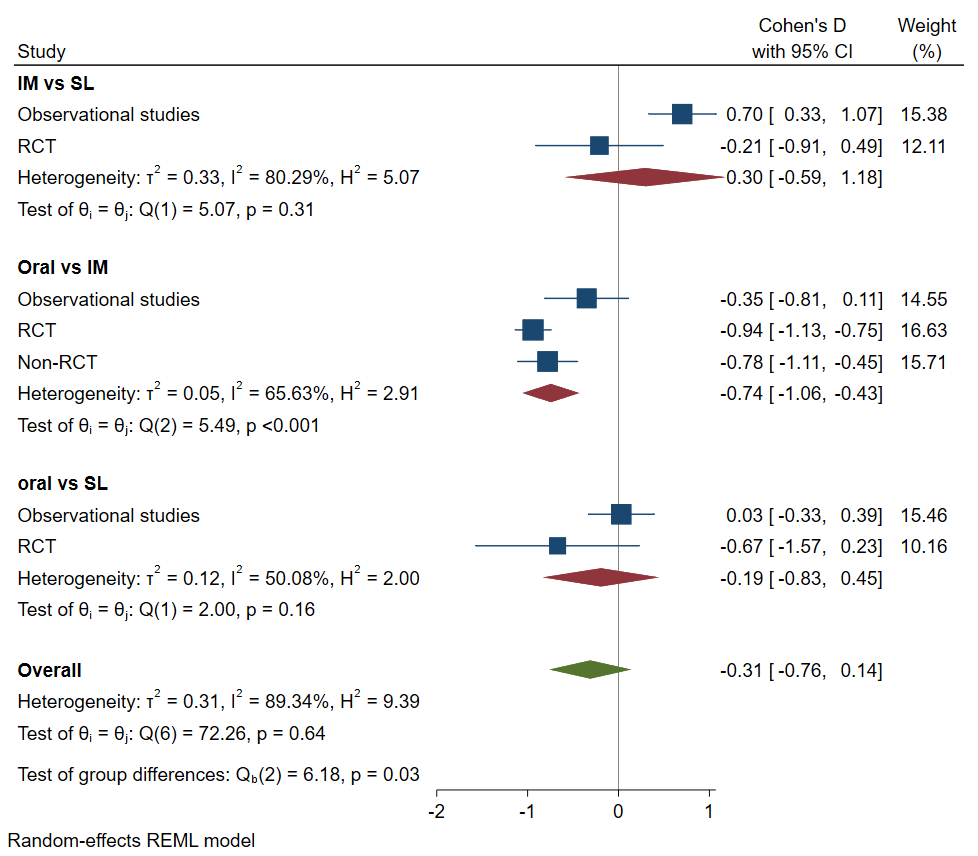


**Figure S3:**


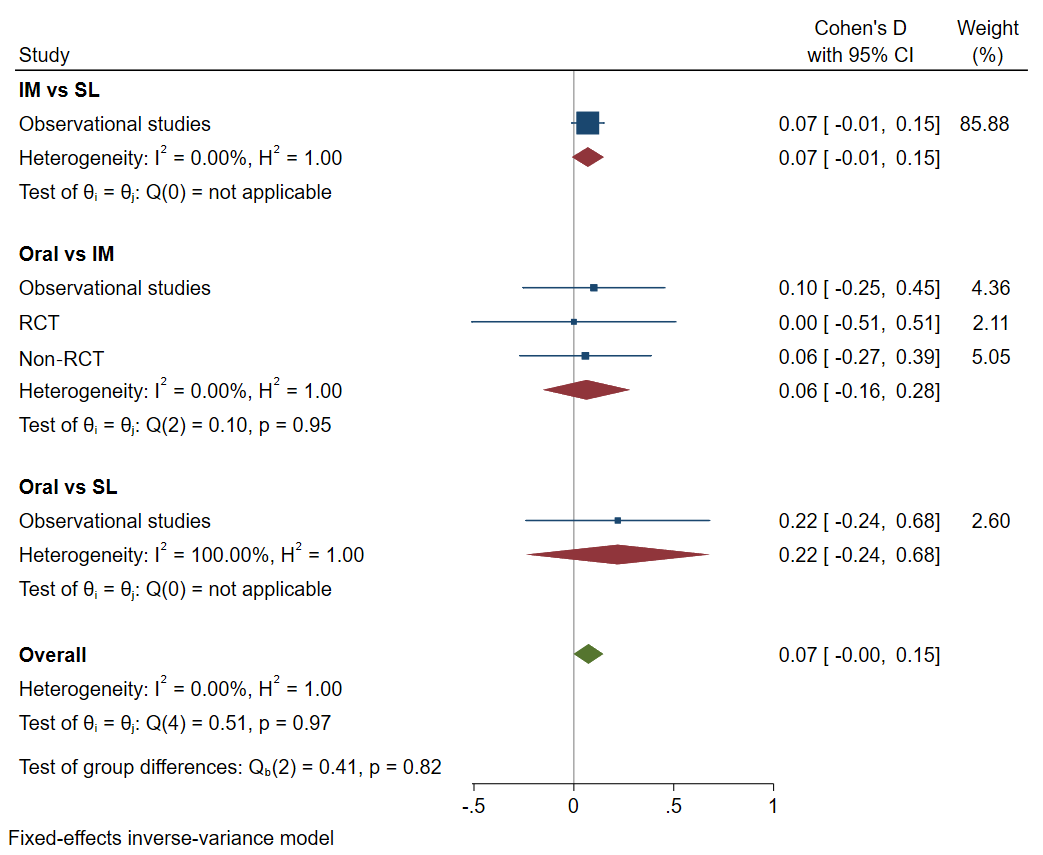


**Figure S4:**

**
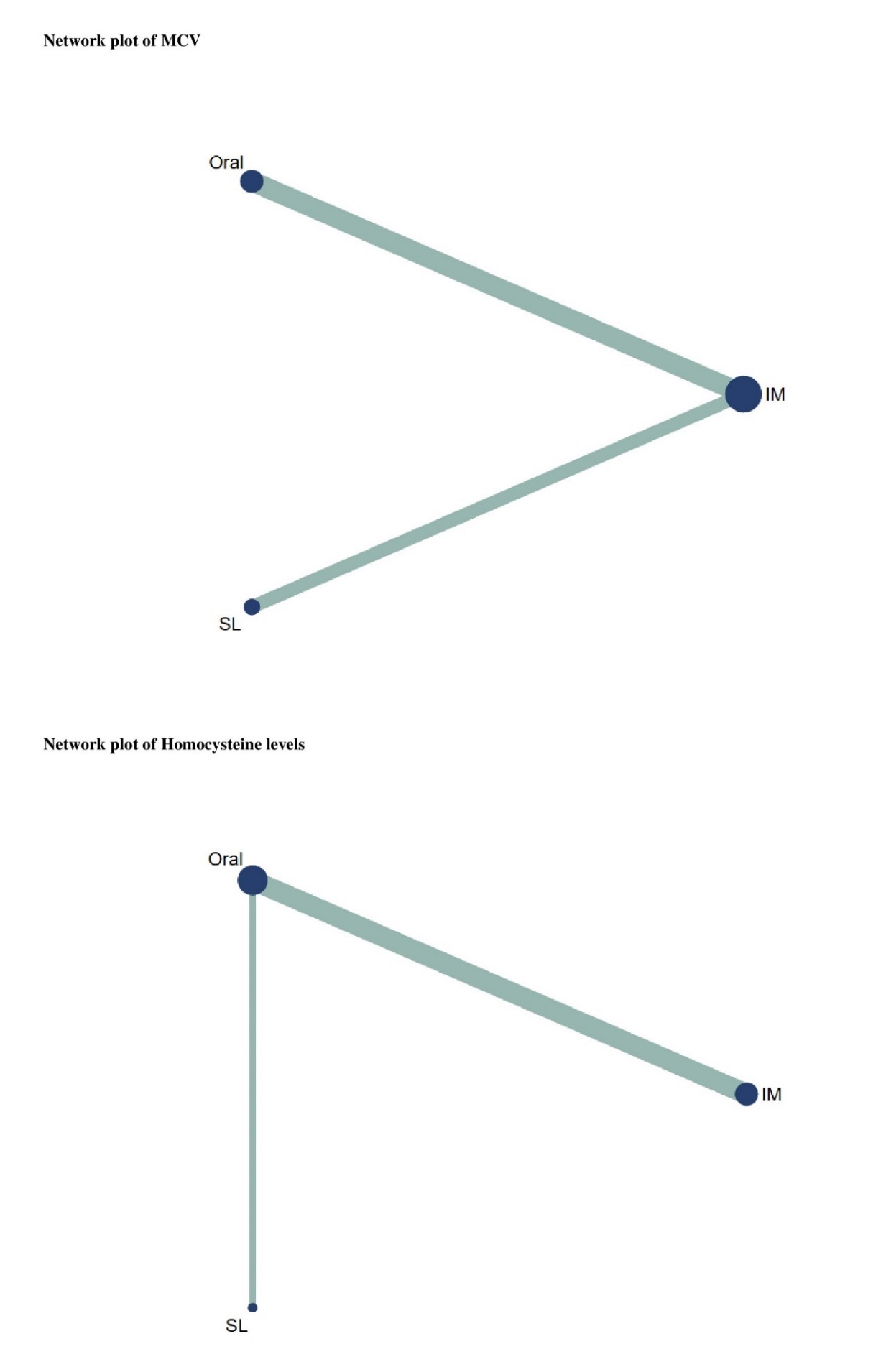
**

**Figure S5:**

**
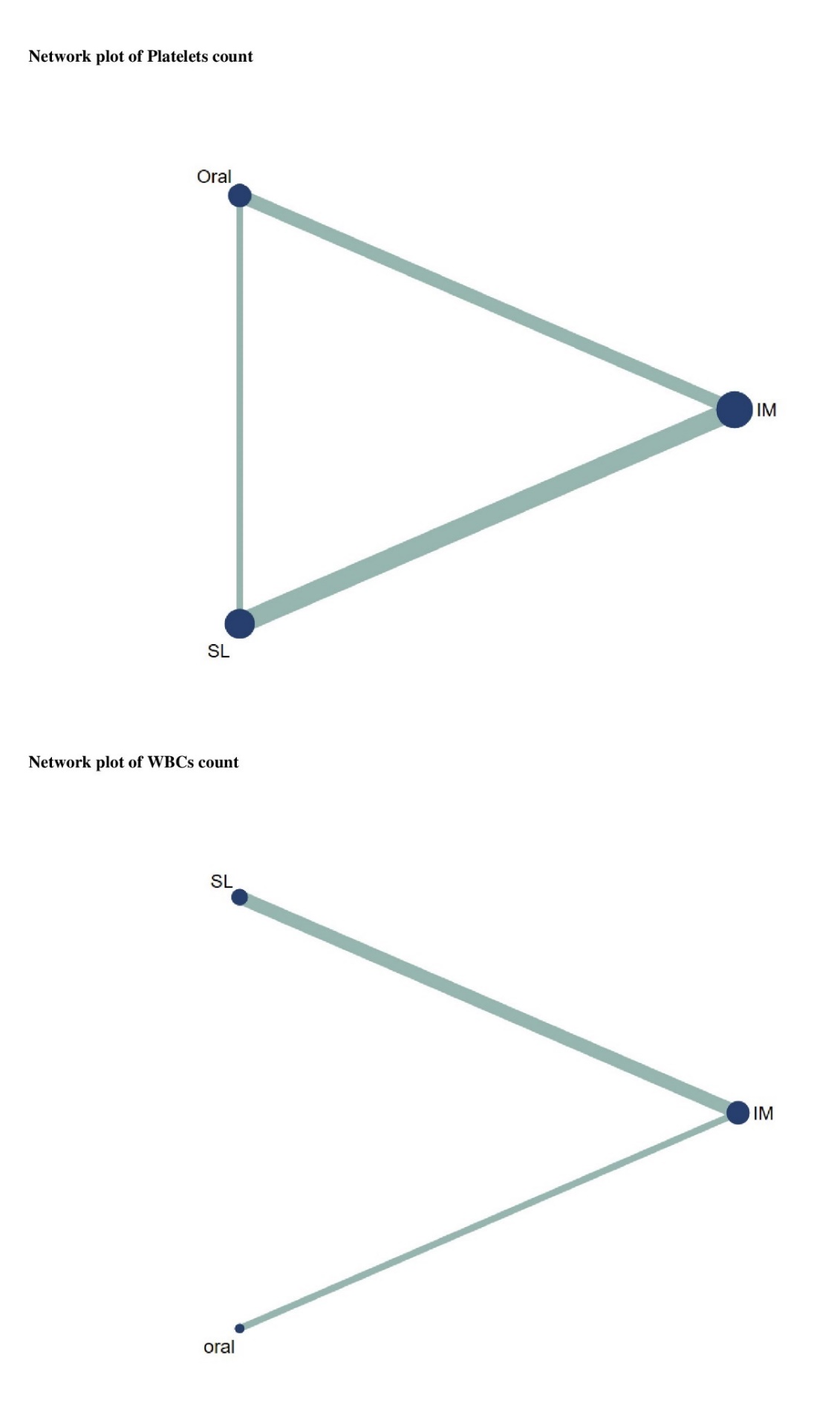
**

**Figure S6:**


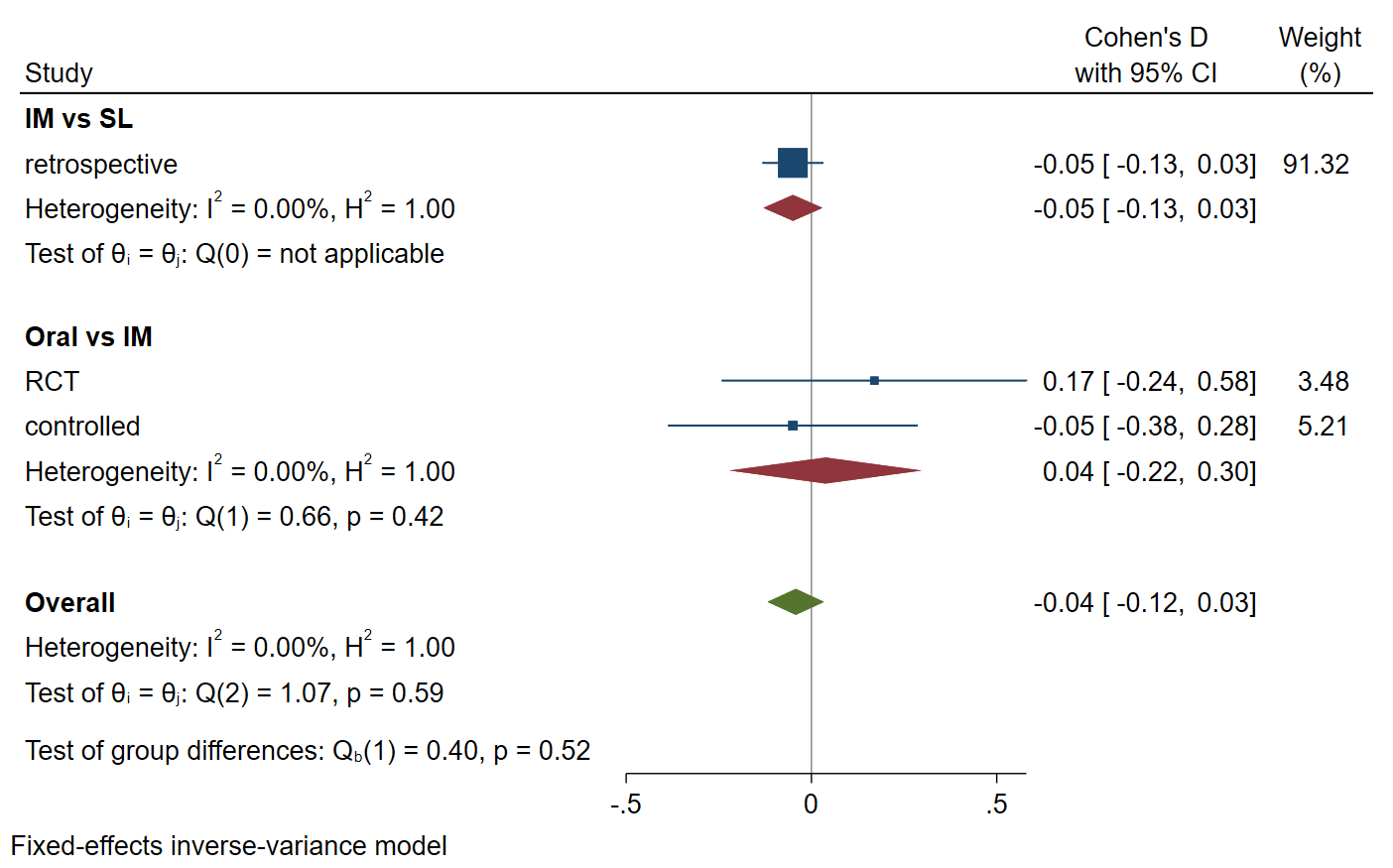


**Figure S7:**


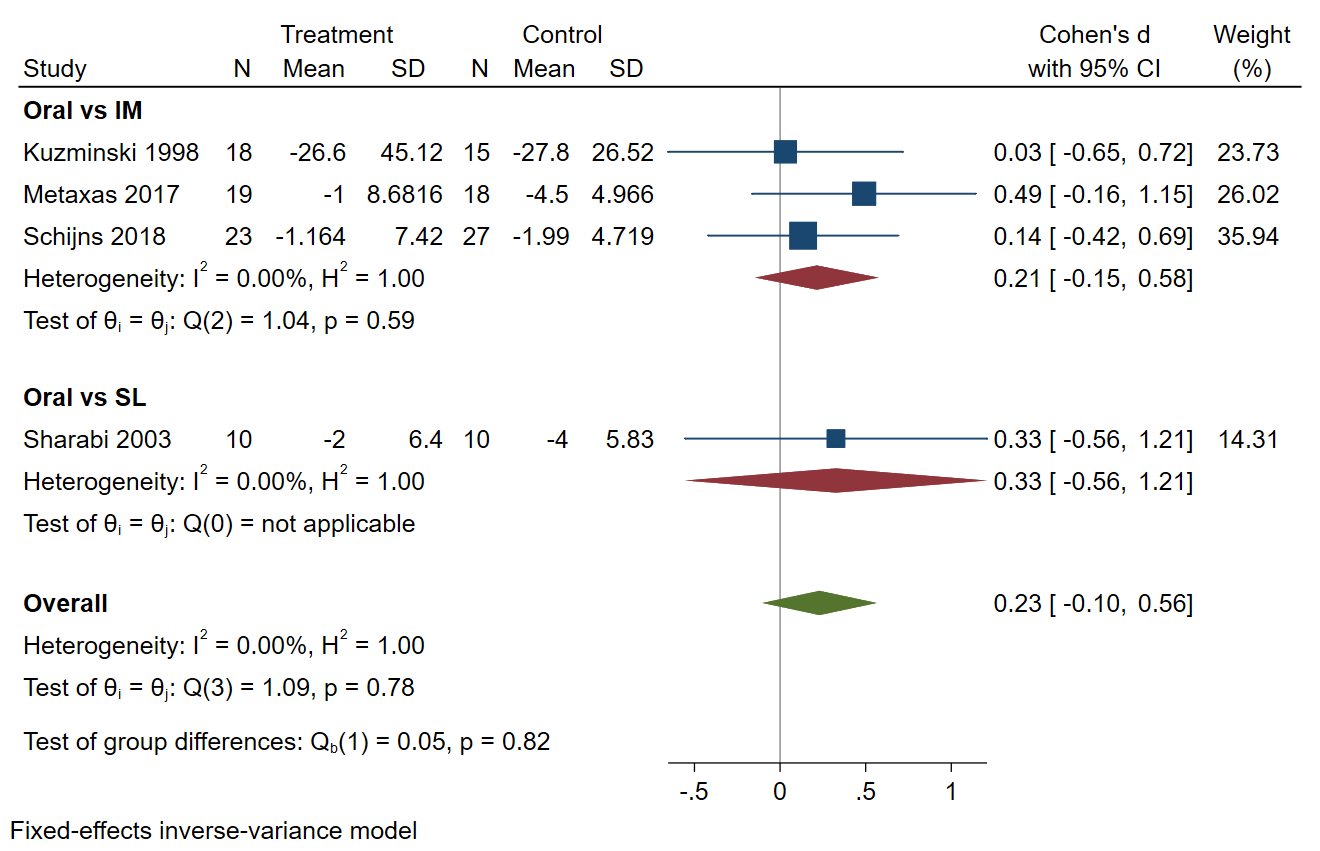


**Figure S8:**


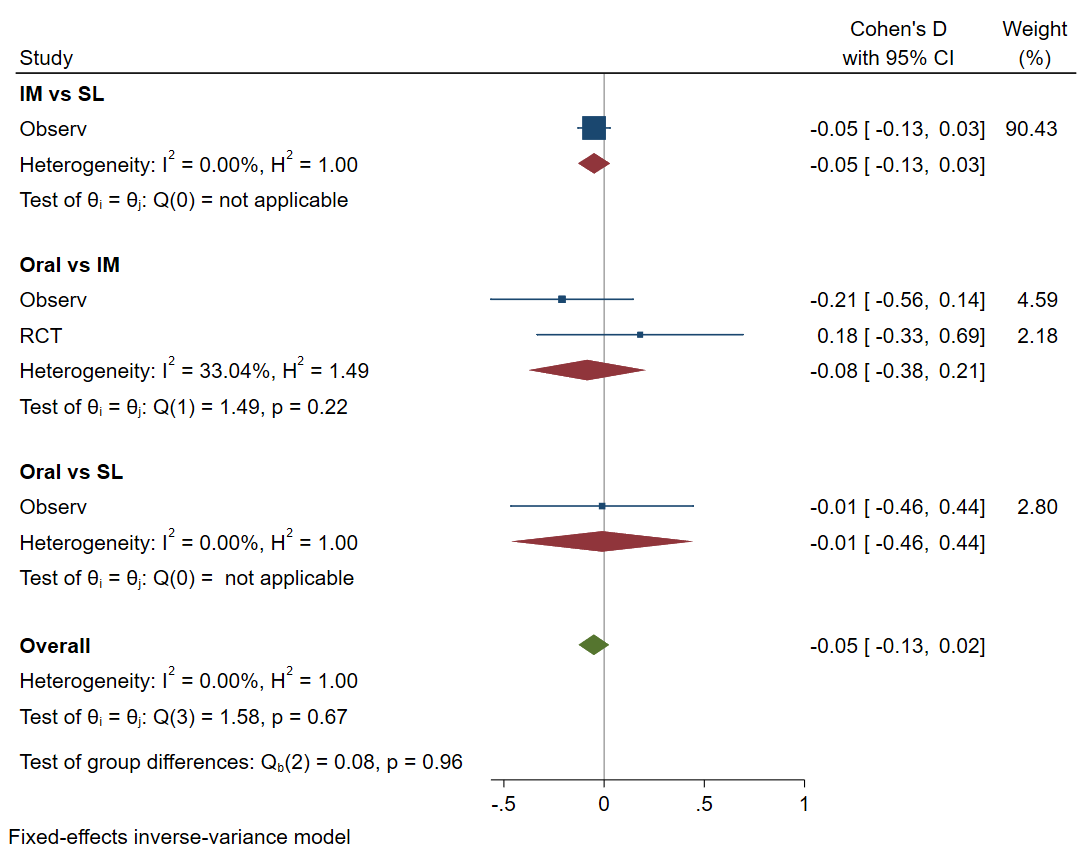


**Figure S9:**


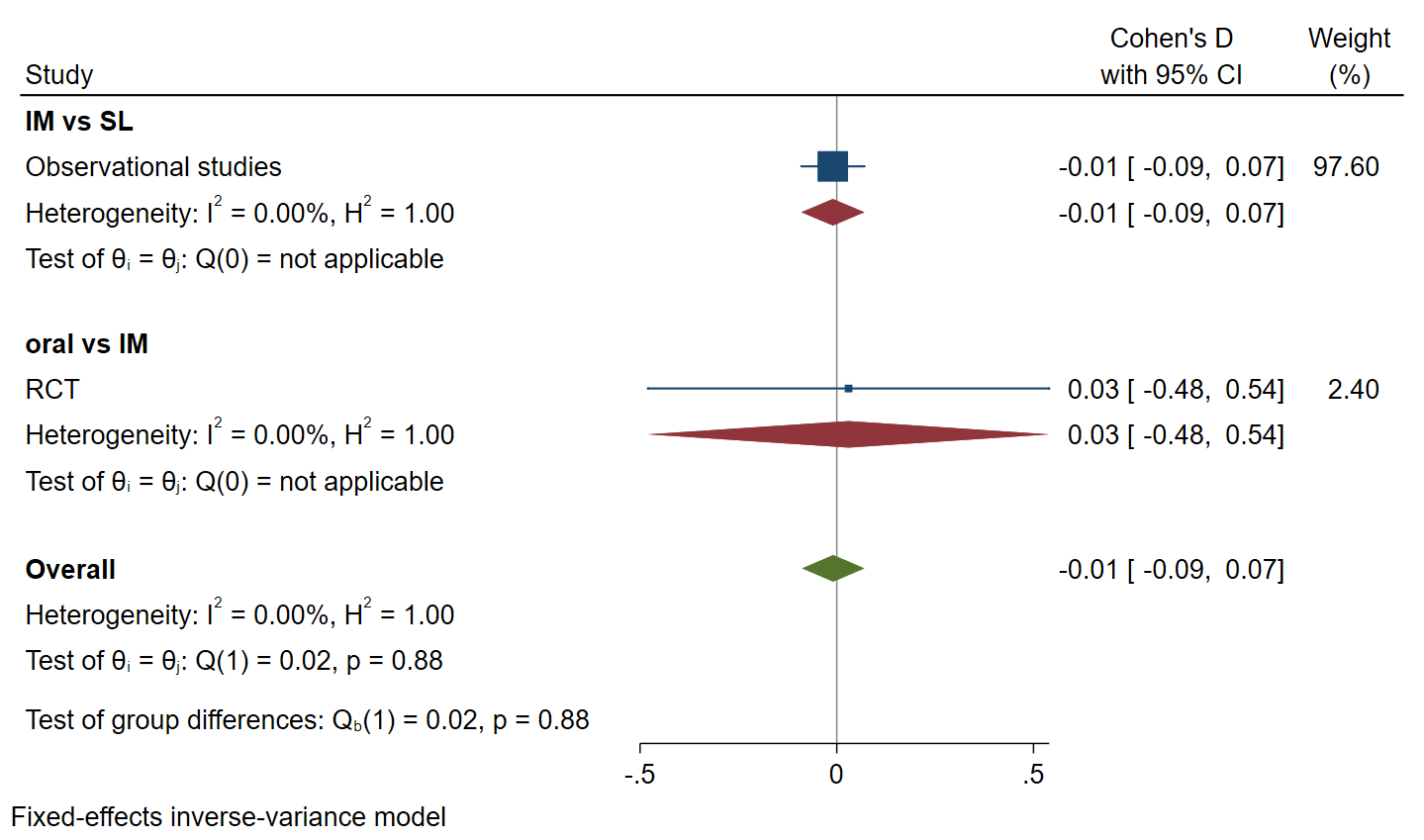


| Treatment by outcome | MCV | Homocysteine | Platelets | WBCs |
| --- | --- | --- | --- | --- |
|  | **SUCRA** | **SUCRA** | **SUCRA** | **SUCRA** |
| oral | 57.7 | 82.7 | 36.6 | 60.5 |
| IM | 27.7 | 32.5 | 50 | 63.5 |
| SL | 64.5 | 34.8 | 63.4 | 26 |

**Table 5: SUCRA of secondary outcomes.**

## Summary of advantages and disadvantages of each route of administration of vitamin B12

***4.3.1 sublingual route of administration***

**Advantages**

It is non-invasive with rapid systemic impact because it allows the drug to be absorbed directly via the mouth's mucosal membrane beneath the tongue and its action begins quickly. Also, rapid absorption occurs due to the enormous vascularization behind the tongue. Bioavailability has improved with fewer side effects. So, Acceptable and effective for patients with nausea, vomiting, schizophrenia, and migraines. Bypassing the GI tract and the hepatic portal system and avoiding hepatic first-pass metabolism increases medication bioavailability. The sublingual region is far more accessible and permeable than the buccal region. Because the pH in the mouth is close to neutral, the medication will be more stable. Easy to administrate the drug. Patient compliance has improved, and there is no pain during administration.[1–4] The SL route of drug delivery utilizes the permeability of the mucosal membrane located on the ventral side of the tongue. Upon SL administration, the drug reaches directly into the bloodstream through the ventral surface of the tongue and floor of the mouth. The primary mechanism for the absorption of the drug into oral mucosa is via passive diffusion into the pilonidal membrane.[3,4] The sublingual membrane is a preventative barrier for permeating many compounds into the systemic circulation. However, as discussed later, this pathway provides several advantages compared to other delivery routes.[4]

**Disadvantages**

Unsuitable for patients who are reluctant to cooperate or unconscious or comatose. Bitter medications should not be used. Compliance may be poor in some patients. It is not allowed to eat, drink and smoke during drug administration. It is not permitted to administer a highly ionic medication. Because it is uncomfortable to hold the dosage in one's mouth, any amount ingested must be considered an oral dose and exposed to first-pass metabolism.[1–4]

***4.3.2 intramuscular route of administration***

**Advantages**

This route is appropriate for administering mild irritants, depot injections, soluble agents, suspensions, and aqueous or oleaginous. IM administration has rapid and uniform drug absorption, particularly in aquatic solutions. In comparison to the oral and subcutaneous methods, this route has a rapid onset of action. Due to the high vascularization of muscles, the medication may be absorbed homologously or through the lymphatic system. Depot injections enable a more gradual, sustained, and extended action. Highly effective in a variety of emergency situations. Through intramuscular injection, the first-pass metabolism is bypassed. [5–8]

Additionally, it overcomes gastrointestinal variables that may affect medication absorption. It has equal efficacy and potency to intravenous drug delivery systems while causing less pain. In comparison to the subcutaneous approach, a greater volume of medication can be delivered.[5–8]

**Disadvantages**

A skilled and qualified individual is necessary to administer the medication via the intramuscular route. Injecting intramuscularly at the correct location may be challenging in kids or people requiring physical restraint. Self-administration of the medication is challenging. It is necessary to maintain aseptic conditions. Intramuscular injections are uncomfortable and painful and may result in an abscess at the injection site. The possibility exists that the drug preparation will deposit improperly in nerves, fats, blood vessels, between muscle fibres, or in sheaths between muscle bundles. [5–10]

There may be nerve damage, resulting in paresis of the muscle supplied by the nerve. Injections performed incorrectly or superficially deposit the solution in the fascia or subcutaneous tissue, causing a later and unpredictable onset. The bulk and vascularity of the muscle determine the drug's absorption. It has prolonged adverse consequences as a result of delayed medication release from the muscle compartment. Precautions should be taken to ensure that the solution is prepared in an adequate amount for the size of the injected muscle, as an increased volume may result in pain, discomfort, tissue injury, and limiting absorption. The drug's onset and duration of effect are not adjustable. Suspensions and greasy medications are not permitted to be administered, and if administered, they may be very painful. It may cause anxiety in the patient, particularly youngsters and kids. Patients must be restrained for a short period, especially if they are children. In the situation of anaphylaxis or neurovascular injury, an intravenous (IV) line must be successfully introduced. Unlike intravenous administration, large volumes cannot be delivered.[5–10]

***4.3.3 oral route of administration***

**Advantages**

It is the easiest, most convenient, and safe route of administering medication. It is suitable for repeated and prolonged usage. Certain drugs with short half-lives are taken orally as sustained-release capsules absorbed over many hours. The duration of activity may extend into the period following therapy. It is painless, self-administered, and requires little training. Oral administration is less undesirable than parenteral administration. Prescribed drugs are widely available. It is cost-efficient since it does not impose additional costs on the patient. There is no requirement for sterile precautions. It does not require specific skills or materials (syringes, needles) to operate. If the medication is solid, such as a pill or capsule, the patient needs just one or two glasses of water, which is easily available. If the medication is in liquid form, all that is required is a measurement instrument, which is typically present with the medication. The risk of an immediate drug reaction is quite low. In general, adverse drug effects are milder.[5,11,12]

**Disadvantages**

It is not ideal for emergencies due to the comparatively slow onset of action of orally taken medications. It is only appropriate for conscious individuals and those who can swallow. It demands the participation or collaboration of the patient, particularly outpatients. It is not recommended for the following drugs: those that are unpleasant and very irritating, those that are destroyed by stomach acid and digestive juices, those that undergo significant first-pass metabolism, patients who have severe vomiting or diarrhoea, and noncompliant patients. The first-pass impact is significant when administering drugs orally. It is a term that refers to the process of drug metabolism in which the concentration of the medication is dramatically reduced before it enters the systemic circulation, most frequently attributed to liver metabolism. Most dosages are empirical. No possibility of titration to a clinical endpoint. Varying absorption results in an unexpected reaction. Ineffective in highly apprehensive patients. Action duration may be prolonged into the posttreatment phase. Drug solubility changes can occur as a result of interactions with other substances found in the gastrointestinal system.[5,11,12]

**References**

[1] Patel P, Makwana S, Jobanputra U, Ravat M, Ajmera A, Patel M. Sublingual route for the systemic delivery of ondansetron. International Journal of Drug Development and Research 2011;3.

[2] Narang N, Sharma J. Sublingual mucosa as a route for systemic drug delivery. Int J Pharm Pharm Sci 2011;3.

[3] Singh M, Chitranshi N, Singh AP, Arora V, Siddiqi AW. An overview on fast disintegrating sublingual tablets. International Journal of Drug Delivery 2012;4.

[4] Pawar PP, Ghorpade HS, Kokane BA. Sublingual route for systemic drug delivery. Journal of Drug Delivery and Therapeutics 2018;8. https://doi.org/10.22270/jddt.v8i6-s.2097.

[5] Raj GM, Raveendran R. Introduction to basics of pharmacology and toxicology: Volume 1: General and molecular pharmacology: Principles of drug action. 2019. https://doi.org/10.1007/978-981-32-9779-1.

[6] Kwatra S, Taneja G, Nasa N. Alternative Routes of Drug Administration- Transdermal, Pulmonary & Parenteral. Indo Global Journal of Pharmaceutical Sciences 2012;2.

[7] Saxen MA. Pharmacologic Management of Patient Behavior. McDonald and Avery’s Dentistry for the Child and Adolescent: Tenth Edition, 2016. https://doi.org/10.1016/B978-0-323-28745-6.00017-X.

[8] Polania Gutierrez JJ, Munakomi S. Intramuscular Injection. StatPearls, Treasure Island (FL): StatPearls Publishing Copyright © 2022, StatPearls Publishing LLC.; 2022.

[9] Sisson H. Aspirating during the intramuscular injection procedure: A systematic literature review. J Clin Nurs 2015;24. https://doi.org/10.1111/jocn.12824.

[10] Una Hopkins, RN, FNP-BC D, Claudia Y. Arias, RN O, Hopkins U, Arias CY, Una Hopkins, RN, FNP-BC D, Claudia Y. Arias, RN O, et al. Large-volume IM injections: A review of best practices. Oncol Nurse Advis 2013;IM.

[11] Talevi A, Quiroga PAM. ADME Processes in Pharmaceutical Sciences (Dosage, Design, and Pharmacotherapy Success). Routes of Drug Administration Chapter 6 2018.

[12] Giovannitti JA, Trapp LD. Adult sedation: Oral, rectal, IM, IV. Anesth Prog 1991;38.
